# Collectanea Medica: Consisting of Anecdotes, Facts, Extracts, Illustrations, &c.

**Published:** 1824-05

**Authors:** 


### COLLECTANEA MEDICA:

CONSISTING OF

### ANECDOTES, FACTS, EXTRACTS, ILLUSTRATIONS, &c.

***Relating to the History or the Art of Medicine, and the Collateral Sciences.***

**Floriferis, ut apes, in saltibus omnia libant,**

**Omnia nos, itidem, depascimur aurea dicta.**

Art. I.—*Extracts from "Medical Jurisprudence."* By J. A. Paris, M.D. &c. and J. S. M. Fonblanque, Esq.

*Syncope;*

In which the pulsations of the heart cease, before the action of the respiratory organs.

The heart may cease to beat either from organic lesions in its own structure, or in that of its vessels; or from being sympathetically affected by injuries in other parts; or from the operation of certain poisons; or from a shock of the general nervous system, as experienced in paroxysms of certain passions.

In ordinary fainting, it is evident that some slight and feeble motions of the heart still continue, although insufficient to produce a sensible pulsation in the more distant arteries; and where this has continued for an unusual period, and the respiration has been so obscure as to escape common observation, the phenomenon has been eagerly seized by the admirers of the marvellous, and credulity has attached to its history, under the name of *trance*, circumstances of extravagance and mystery, to which it can hardly be necessary to allude on the present occasion. But the motions of the heart may have ceased altogether; and in such cases it becomes a question, no less interesting to the practical physician than to the physiologist, whether they can ever be restored? and, if so, we have to inquire under what limitation as to time, under what circumstances, and by what means? The views which have been already offered respecting the pathology of syncope, will afford us considerable assistance in the solution of a problem so intimately connected with inquiries of forensic importance. It would appear that, where the heart has ceased to pulsate in consequence of the cessation of respiration, it can never again be set in motion; but that, where it has stopped from other causes, as from the operation of certain poisons, its muscular irritability not having been exhausted, its action may be occasionally revived. Where syncope arises from hemorrhage, we shall find, on dissection, that the heart and its great vessels are either empty, or contain only a small quantity of blood in their cavities; but, where syncope arises from other causes, the heart is seen distended to an unusual magnitude, and the blood in the left auricle and ventricle is generally of a more or less florid colour, and has not the hue of venous blood; a circumstance which depends upon the pulsation of the heart ceasing before the function of respiration, and which is the very reverse of what happens in death from suffocation, as we shall hereafter explain.

Violent passions of the mind very commonly produce syncope, which has in some instances terminated in death: we are, however, inclined to believe that, in fatal cases of this nature, the persons must have laboured under some organic affection of the heart, or its vessels. Philip V. died suddenly on being told that the Spaniards had been defeated, and, on opening the body, his heart was found ruptured.

Dr. Tissot relates also the case of the father of a numerous family, who, having lost his wife whom he tenderly loved, was suddenly seized with laborious respiration, and died at the end of two days; when the lungs were found gorged with blood, and the heart ruptured. Now, in both these cases, it is probable that the muscular structure of the heart had been softened by previous disease. So in the case of Mr. John Hunter, whose life was suddenly extinguished by mental emotion, the valves of the heart had been long in a state of disease; and, so well aware was he of the danger to which he was constantly exposed, that he had, for some time previous to his death, been in the habit of retiring from all those situations in which his passions were likely to be excited. It is said that the instances of death from sudden joy are more numerous than those from grief, probably because the effect of this latter passion is rather to retard than to accelerate the circulation. Sophocles, being desirous of proving that at an advanced age he was in full possession of his intellectual powers, composed a tragedy, was crowned, and died through joy. The same fate befel Philippides, the comic writer: thus, too, the Lacedemonian Chilon expired in the embrace of his son, who had borne away the prize at the Olympic games; and we read of Roman women who died in the same manner, upon seeing their sons return from the battles of Thrasymene and Cannæ. On the other hand, we might adduce much classical authority to show that death has frequently been the sudden effect of grief.

Montaigne relates the case of a German, who, after having performed great feats of valour, was killed at the siege of Osen: one of the general officers, having desired to see the corpse of so gallant a man, was conducted to the body, when he instantly recognised the features of his own son, and died on the spot. The record of our own times will furnish us with an instance in which an actor of celebrity suddenly expired upon repeating a passage that contained a fancied allusion to the domestic affliction under which he was suffering.

Dr. Ozanam, in illustration of the influence of pain and terror in producing sudden extinction of life, relates the case of a middle-aged criminal, who, having throughout evinced extreme weakness and depression, expired in his way to the scaffold, and was stiff before he arrived at the place of execution, which was about seven miles distant.

In such cases of sudden death, from the operation of violent mental emotions, we apprehend that dissection will frequently demonstrate the existence of previous disease in some of the organs immediately essential to life; and we shall hereafter have occasion to refer to the influence of the passions in hastening the fatal termination of a chronic disease. On the present occasion, we introduce the following extremely interesting case, in confirmation of the position we are endeavouring to maintain: the case was originally published in the Transactions of the Physico-Medical Society of New York, by Dr. Valentine Mott; it afterwards appeared in the Journal Universel des Sciences Medicales, Avril 1819; and lately it has found its way into the Medical Repository of this country. A robust and plethoric female, aged twenty-two, long addicted to dissolute and intemperate habits, had complained for some time of slight and apparently rheumatic pains; but, within a day or two of the fatal event, she had been deserted by a man to whom she was engaged in marriage, in consequence of which her mind became very deeply affected. After having supped on the preceding night, she retired to rest as usual, and in the morning was found dead in bed: she lay in a bent position on the left side, and was hence supposed at first to be in a profound sleep; neither the countenance nor the limbs were in the least distorted. On dissection, the pericardium was found to contain ten ounces of coagulated blood, and two of serum; the heart, on all sides being covered by it, was of ordinary volume, but much loaded with fat. At the summit of the aortic ventricle was discovered the breach from which the effused blood had issued; the parietes of the ventricle around the rupture were much thicker than in the natural state, and, on close examination, a very sensible fluctuation was distinguished, to the extent of an inch on one side of it, from which flocculi, of a cheese-like substance, were discharged on pressure. The pericardium also presented traces of inflammation.

We have here, then, a case in which a morbid change in the structure of the heart had existed for a considerable period, and which was suddenly brought to a fatal termination by an affection of the mind.

Before we quit the consideration of syncope, we have to notice a fatal variety of that disease, which well deserves the attentive consideration of the forensic physician, whose highest duty, let it be remembered, is the investigation of sudden death. It is described by Mr. Chevalier under the term *asphyxia idiopathica*, in which the patient suddenly faints and dies: the essential circumstances of the disease evidently denote, says Mr. Chevalier, a sudden loss of power in the extreme vessels to propel the blood; in consequence of which the heart, after having contracted so as to empty itself, and then dilated again, continues relaxed for want of the return of its accustomed stimulus, and dies in that dilated state. On dissection, all the cavities of the heart are found completely empty, and the viscus itself in a state of extreme flaccidity.

### *Suffocation.*

Suffocation may be defined, the destruction of life by the suspension of the function of respiration, occasioned by external violence. Unless we add "*by external violence,*" we shall perceive that the definition would be far too comprehensive; and the term *suffocation* would be made to embrace a much wider range of subjects than its popular acceptation would allow. If the physiological views be correct which we have adopted and explained in the foregoing section, "on the Causes and Phenomena of Sudden Death," we should be compelled, without such a protecting adjunct, to include under the history of suffocation, not only the phenomena of drowning, strangling, hanging, smothering, and noxious inhalation, but even those of apoplexy, fatal intoxication, and various diseases of the brain and spinal marrow, together with the effects of a great proportion of poisons; for, by such agents, death is undoubtedly occasioned through the failure of the respiratory functions.

In death from suffocation, the heart continues to pulsate for several minutes after the breathing has entirely ceased, in consequence of which the blood which passes through the pulmonary vessels no longer receives the influence of oxygen, and therefore *black* blood circulates: the brain, it would appear, soon feels the want of the florid arterial stream, by which alone its energies can be maintained. Bichat has shown that, when dark-coloured blood is injected into the vessels of the brain, by means of a syringe connected with the carotid artery, the functions of the brain become immediately disturbed, and in a short time entirely cease: the effect is precisely similar, whether the dark-coloured blood be transmitted to the brain by the syringe of the experimentalist, or by the heart itself. It is not until after the full effects of the suspended respiration are thus produced on the brain, that the motions of the heart become enfeebled, and that the ventricles contract less powerfully and at longer intervals: at length, the action of the heart is altogether arrested, and, if the thorax be examined at the instant that the circulation has ceased, nothing is observed except a slight tremulous motion of the auricles. The cavities of the left side are much contracted, and contain only a small quantity of blood; while the right auricle and ventricle, and the large vessels communicating with them, are distended to an unusual size. This state of the heart, it will be observed, is very different from that which we have described as constantly occurring after syncope. In the contemplation of these phenomena, a question very naturally suggests itself in regard to the probable interval which elapses between the cessation of respiration and the consequent failure of the heart's action: in other words, it may be asked, how long can the heart support its contractions without the aid of respiration? It would appear that this interval not only varies in duration in different animals, but even in the same animal under different circumstances,—such as that of age, capacity of the thorax, quantity of air in the lungs, state of the stomach, and general vigour of the animal; but in man, under the most favourable circumstances, it is extremely doubtful whether the heart ever continues to pulsate for so long a period as five minutes after the lungs have ceased to perform their office; and it is very questionable whether, in most instances, the interval is not considerably shorter than this.

1. *By Drowning.*—It was formerly believed that asphyxia from drowning always depended upon the lungs and intestinal canal being filled with water; whereas, it is hardly necessary to observe that it alone depends upon the blood, in consequence of the suspension of breathing, ceasing to possess the qualities which are essential to the preservation of life. M. Gauteron immersed a dog for more than a quarter of an hour without inflicting the least injury, having previously inserted a long tube in the trachea, which was kept elevated during the experiment above the surface of the water.

If a small animal be immersed in water contained in a transparent glass vessel, the phenomena of drowning are readily discernible. There is first a deep expiration, by which bubbles of air are expelled from the lungs; there is then an effort to inspire, but the effort is ineffectual; there being no air which can be received into the lungs, and a spasm of the muscles of the glottis seems to forbid the admission of any considerable quantity of water into the trachea. The attempts to breathe are repeated several times, and at each attempt at expiration a small proportion of air is expelled from the mouth and nostrils, until the air-cells of the lungs are almost emptied; then the animal becomes insensible, and convulsive action of the voluntary muscles mark the instant when the brain begins to suffer from the influx of the dark-coloured venous blood. After the cessation of these convulsive actions, the animal becomes motionless, and gives no sign of life; but, if the hand be applied to the thorax, the actions of the heart, gradually becoming fainter and fainter, indicate that some remains of vitality still linger in the system. Before the circulation of the blood altogether ceases, the muscles of respiration once more resume their actions, and ineffectual efforts are made to breathe. It is a remarkable circumstance that the diaphragm continues to exert itself nearly as long as the heart itself, and that the interval between the cessation of the motions of the diaphragm and that of the motions of the heart, which is so short in animals that die by strangulation, is still shorter in those who perish by drowning. These phenomena follow each other in rapid succession, and the whole scene is closed, and the living animal is converted into a lifeless corpse, incapable of recovery, in the brief space of a few moments. *(Brodie's manuscript Notes.)* If, however, the animal be taken out of the water before the total extinction of life, and the diaphragm contract afterwards, so as to draw air into the lungs before the action of the heart has ceased, the circulation is maintained, and the animal continues to respire: he will thus have escaped immediate death from suffocation; but his life still remains in jeopardy, for there is a second period of danger, and one at which death may take place when we are the least prepared to expect it; for the dark-coloured blood, which has been transmitted through the circulatory system during the suspension of respiration, would seem to act like a narcotic poison upon the brain: no sooner, therefore, does it enter that organ, but deleterious effects are produced, —the animal at first falls into a state of stupor, the pupils of the eyes become dilated, the respiration laborious, the muscles of the body convulsed, and the animal dies, *poisoned by its own blood.*

The body of a person who has died from drowning exhibits a physiognomy which it is important to notice. The whole surface is distinguished by a remarkable coldness and pallor; the eyes are half open, and their pupils considerably dilated; the tongue is pushed forward to the internal edges of the lips, and sometimes wounded; and the mouth and nostrils are covered with foam. At other times, instead of a pallid visage, we have one that is swelled, and bloated with livid blood.

Upon dissection, we shall perceive the vessels of the brain more or less gorged with blood; in the trachea a watery and bloody froth will be found; the lungs will appear expanded, full of frothy mucus, and generally livid; the right cavities of the heart gorged with blood, the left nearly empty; and it has been sometimes noticed that the blood remains fluid, and follows after every incision by the scalpel. The stomach will generally be found to contain some water. Hebenstreit also states that, since in the act of drowning the person dies on an inspiration, the diaphragm is necessarily found convex, or bent towards the abdomen: this statement, however, is erroneous.

Upon these appearances we have a few observations to offer, especially as they have given origin to some important questions; and first with respect to the presence of water in the stomach and lungs, than which few indications, connected with the subject of drowning, have given occasion to greater controversy. For since it hath been observed that water is rarely found in the stomach or lungs of a person who has been submerged after death, it was inferred that the presence of that fluid in these organs necessarily proved that the individual must have been plunged into the water during life. As a general proposition this may be admitted as correct, although it is liable to certain exceptions, with which the medical jurist ought to be acquainted: we may, for instance, suppose a case, in which the submerged person may be so plunged at once under water, as to have been suffocated without his previously coming to the surface; and, when asphyxia has taken place, the powers of deglutition, on which the presence of water in the stomach wholly depends, are at an end; or we may suppose that the party in question faints from terror. A remarkable instance of this kind is quoted by Foderé, from Plater, of a young woman, who, having been condemned to be drowned for infanticide, fainted at the moment she was plunged in the water, and, having remained for a quarter of an hour under its surface, recovered after being drawn out.

With respect to the presence of water in the bronchiæ and lungs, we may observe that, in the violent struggles of a drowning man, a certain portion of water generally passes the epiglottis; and, being immediately mixed with the air and mucus of the trachea, constitutes that frothy mucus which we have described as being so highly characteristic of this species of violent death; although we are not to conclude, with Larrey, that it is the immediate cause of dissolution in such cases. The quantity of water, however, thus forced into the pulmonary structure, is extremely small, for its entrance is powerfully opposed by a spasm of the muscles of the glottis: were it to occur in any considerable quantity, and to appear in its fluid state, instead of that of froth, the inference would clearly be, that it had passed in after death.

Although the presence of this frothy matter must be considered as a strong presumptive proof that the person found in the water had perished by drowning, the converse of this proposition is by no means established by the absence of such an indication.

*The buoyancy of the human body* is another point in the history of drowning, which has occasioned much discussion; and in solving the problem, so highly important in its forensic relations, whether a body found in the water had been drowned, or thrown in after death, it has been considered by some physiologists as capable of affording a certain degree of presumptive evidence, although we are inclined to attach but little or no importance to such an indication. The specific gravity of the human body, under ordinary circumstances, is very little greater than that of fresh water; so small, indeed, is the difference, that, when the lungs are inflated, a man will float with little or no effort, if he have sufficient self-possession, and does not attempt to raise too great a portion of his body out of the sustaining fluid: but, when the air of the lungs is expelled, and probably at the same time a certain quantity of water is taken into the stomach, the body becomes specifically heavier, and the victim sinks. It may be assumed as a general rule, that no newly-drowned body floats, although many facts have been adduced in support of a contrary opinion. The naval custom of loading the dead bodies with weights before they are consigned to a watery grave, is not for the purpose of sinking the corpse, but for preventing its rising after the process of putrefaction has commenced. The period during which a body will remain at the bottom, cannot be very accurately determined, as the change does not take place until a sufficient quantity of air be generated to buoy it again to the surface. In the melancholy instance of the loss of the Royal George, the dead bodies were observed ascending to the surface of the sea on or about the fifth day. The general position of a body which has thus risen, provided there be no external or adventitious circumstances to change it, is such, that it floats nearly immersed, the face, arms, and legs hanging downwards, and the loins being uppermost: this is the form which the body must mechanically and hydrostatically assume, if the sustaining power of generated air be, as it generally will, in the cavity of the abdomen, where putrefaction is more likely to commence; for the head and limbs are generally specifically heavier than water, while the trunk, especially if inflated with air, is somewhat lighter.

It has been said that a position, different from that which we have just described, will take place where the person has been strangled, and the body then thrown into the water; for, in this latter case, it is contended that the lungs will be distended with air, and that, consequently, the sustaining power must be in the thorax. In support of this opinion, the story of the appearance of Caraccioli, admiral of the Neapolitan navy, has been ingeniously adduced. This unfortunate man was hanged in pursuance of the sentence of a court-martial, and his body was committed to the deep in the usual manner; thirteen days after which, while the King of Sicily was walking on the deck of Lord Nelson's ship, he suddenly exclaimed, with a yell of horror, "*Vien! Viene!*" The admiral's corpse, breast high, was seen floating towards the ship; the shot that had been attached to the feet, for the purpose of sinking it, not being sufficiently heavy. This may, perhaps, be explained by supposing that the corpse was stiff before it was immersed; in which case, the centre of gravity being exceedingly low, on account of the shot tied to the feet, he must have floated upright, wherever the buoyant power from generated air might be situated. At all events, we feel no hesitation in at once rejecting the proposition, in support of which it has been brought forward. The fact is that, in relation to gaseous contents, the lungs are the same in strangled as in drowned persons; for in both cases a quantity of air is forcibly expelled from them before dissolution.

